# Mangrove Crab *Ucides cordatus* Removal Does Not Affect Sediment Parameters and Stipule Production in a One Year Experiment in Northern Brazil

**DOI:** 10.1371/journal.pone.0167375

**Published:** 2016-12-01

**Authors:** Nathalie Pülmanns, Ulf Mehlig, Inga Nordhaus, Ulrich Saint-Paul, Karen Diele

**Affiliations:** 1 Department of Ecology, Mangrove ecology group, Leibniz Center for Tropical Marine Ecology, Bremen, Germany; 2 Laboratório de Botânica, Universidade Federal do Pará, Bragança, Paulista, Brazil; 3 School of Applied Sciences, Edinburgh Napier University, Edinburgh, United Kingdom; 4 St Abbs Marine Station, St Abbs, Berwickshire, United Kingdom; Griffith University, AUSTRALIA

## Abstract

Mangrove crabs influence ecosystem processes through bioturbation and/or litter feeding. In Brazilian mangroves, the abundant and commercially important crab *Ucides cordatus* is the main faunal modifier of microtopography establishing up to 2 m deep burrows. They process more than 70% of the leaf litter and propagule production, thus promoting microbial degradation of detritus and benefiting microbe-feeding fiddler crabs. The accelerated nutrient turn-over and increased sediment oxygenation mediated by *U*. *cordatus* may enhance mangrove tree growth. Such positive feed-back loop was tested in North Brazil through a one year crab removal experiment simulating increased harvesting rates in a mature *Rhizophora mangle* forest. Investigated response parameters were sediment salinity, organic matter content, CO_2_ efflux rates of the surface sediment, and reduction potential. We also determined stipule fall of the mangrove tree *R*. *mangle* as a proxy for tree growth. Three treatments were applied to twelve experimental plots (13 m × 13 m each): crab removal, disturbance control and control. Within one year, the number of *U*. *cordatus* burrows inside the four removal plots decreased on average to 52% of the initial number. Despite this distinct reduction in burrow density of this large bioturbator, none of the measured parameters differed between treatments. Instead, most parameters were clearly influenced by seasonal changes in precipitation. Hence, in the studied *R*. *mangle* forest, abiotic factors seem to be more important drivers of ecosystem processes than factors mediated by *U*. *cordatus*, at least within the studied timespan of one year.

## Introduction

Burrowing crabs are ecosystem engineers and their importance for sediment processes has been discussed for many years [1–5], along with effects of their feeding activities on forest structure and nutrient cycling [2,6–11]. To determine the ecological roles and ecosystemic importance of burrowing crabs, addition or exclusion and removal experiments have been performed. In such experiments the size of the experimental plots varies depending on the size and density of the crabs and the underlying research questions. For example, for determining the effects of exclusion of small fiddler crabs (*Uca* spp.) on the growth of mangrove seedlings, small scale experiments with plot sizes of 1 m × 1 m proved to be sufficient [12]. Assessing the effects of larger crabs in mature forests requires larger plot sizes and longer experimental time frames, and thus considerably more effort. Only one such study, excluding crabs for 12 months from three 225 m^2^ plots, has been performed to date in Australia [5].

Exclusion of burrowing fiddler crabs from salt marshes led to an increase in meiofaunal density, probably due to reduced competition for food (bacteria, microphytobenthos) on the sediment surface [13,14]. In other studies, however, a reduction of crab burrows decreased the density of associated meio- and macrobentic infauna [15,16]. Reduced bioturbation can result in more saline and reduced sediment conditions and also affect the growth of mangrove seedlings [12,17]. Removal of bioturbating fiddler crabs also decreased microbial activity, fungal decomposition and leaching rates of organic matter in the upper sediment layer of salt marshes [18,19]. Similarly, crab removal in an Australian mangrove forest, and the only experiment to date with larger plot sizes, resulted in increased concentrations of sulfide and ammonium in the sediment and decreased leaf production of trees, as indicated by a significantly reduced stipule fall rate [5].

Most past research efforts to investigate the ecosystemic role of crabs have focussed on smaller burrowing species and small-scale experiments, and on crabs from the Indo-West-Pacific (IWP). Results from the IWP region are not necessarily representative for the Atlantic-east-Pacific (AEP) system, since flora and invertebrate fauna of the latter biogeographical area is much less diverse [20].

On the Atlantic side of the AEP, mangrove microtopography is dominated by the large burrowing crab *Ucides cordatus* (Ucididae). The species is an obligate mangrove dweller, living in up to 2 m deep burrows [9]. In Northern Brazil, *U*. *cordatus* occurs at average densities of 1.7 individuals m^-2^ [21]. Due to its large size (carapace width up to 10 cm [22]), it provides 63% of the total faunal biomass compared to sympatric fiddler crabs, which contribute 12% with approximately 19 individuals m^-2^ [7]. *U*. *cordatus* sustains its high biomass by processing more than two thirds of the annual mangrove litter and propagule production in the high intertidal N-Brazilian forest, most of which would otherwise be exported by the tides [8,9]. However, since *U*. *cordatus* assimilates only parts of the energy inherent in the food, a large percentage of the matter remains as leaf fragments, due to sloppy feeding, or as faeces, becoming available for decomposing bacteria.

Koch and Wolff [7] hypothesized for this system that fiddler crabs feed on bacteria, which in turn feed on the leaf remains from *U*. *cordatus*. They further postulated a positive feedback effect on the primary production, since nutrients in the leaf litter are first retained by the feeding activity of *U*. *cordatus* and then re-mineralized by bacteria, thereby becoming available to the trees. The authors additionally hypothesized that a significant reduction or loss of one of the model's components would have a negative impact on the remaining components, consequently affecting primary production If, for example, crab numbers decreased significantly in an otherwise healthy mangrove forest, nutrients locked in leaf litter would no longer be retained in the system under this scenario. Mangrove trees would suffer from decreased bioturbation, since e.g. the crabs’ burrows facilitate sediment oxygenation, preventing the formation of phytotoxins such as H_2_S [7]. Burrowing crabs may also impact sediment desalination, the reduction state, the organic matter decomposition and thereby CO_2_ efflux rates of the sediment, which in turn may drive changes in stipule production.

No manipulative experiment has been conducted yet to assess the above predictions. It is however important to understand the functional role of *U*. *cordatus* in the mangrove ecosystem, given re-occurring crab population declines in North-eastern Brazil caused by a spreading fungal disease (“Lethargic crab disease” [23,24]) and increasing fishing pressure across the country due to the application of a new illegal capture technique [25]. *U*. *cordatus* is economically important, providing the livelihood for thousands of artisanal fishermen in Brazil. In Northern Brazil, approximately 7 tons per km^2^ are captured per year [26]. Due to its slow growth [27,28] the species is listed in Brazilian legislation under the category “species at risk of overexploitation or overfished” [29].

We have conducted a one year *U*. *cordatus* removal experiment in the mangrove forest of the Northern Brazilian Caeté estuary to investigate possible ecosystemic effects of significantly decreased crab numbers, such as would result from a significantly increased fishing pressure. The following sediment parameters were monitored: salinity, organic matter content, CO_2_ efflux rate of the surface sediment (as a proxy for microbial carbon degradation) and reduction potential. To assess impacts on biota, tree leaf production using stipule fall as proxy [27] were assessed.

We hypothesize that a reduction of the number of *U*. *cordatus* leads to 1) increasing sediment salinity, 2) decreasing organic matter content of the sediment, 3) reduced CO_2_ efflux rates of the surface sediment due to a decrease in organic matter and 4) more reduced conditions in the sediment. Further, we predict that the reduction of *U*. *cordatus* will 5) decrease stipule production.

## Material and Methods

### Study area

The study was performed in a mangrove forest in the Caeté estuary, Pará state, North Brazil. Field work permission was granted by the Instituto Chico Mendes de Conservação da Biodiversidade (ICMBIO) and the Stakeholder Council of the Extrativist Reserve Caeté-Taperaçu, SISBIO Authorization number: 30007–1. The removal experiment was implemented near the tidal channel Furo Grande on the Ajuruteua peninsula (46°38’W 0°50’S). At the study site, the dominant mangrove tree species is *Rhizophora mangle* L. (Rhizophoraceae). Other mangrove tree species are *Avicennia germinans* (L.) L. (Acanthaceae) and *Laguncularia racemosa* (L.) C. F. Gaertn. (Combretaceae) [30].

The region has semidiurnal tides with amplitudes of 2 to 5 m [31,32]. Mean annual temperature for the study years 2011 and 2012 was 26.1°C (Tracuateua weather station, 50 km from the study site). Precipitation was 2621 mm in 2011 and decreased to 1552 mm in 2012 [33]. The wet season occurs typically from January to August and the dry season (monthly precipitation < 100 mm) from September to December [32].

### Experimental design

Twelve experimental plots (13 m × 13 m) were established in the high intertidal zone containing exclusively *R*. *mangle* trees (6–18 trees per plot, up to 15 m height), with a minimum distance of 15 m in-between them. To reduce variability, plots with similar inundation frequencies were chosen, as indicated by height range of algal growth on the trees' stems, and visually similar sediment characteristics. Each plot was placed around a central mature *R*. *mangle* tree. The diameter of central trees (measured in the cylindrical portion of the stem 50 cm above the highest stilt root) ranged between 18 and 26.5 cm (mean ± standard error: 21.7 ± 0.8), central tree heights ranged between 10–14 m. All above ground stilt roots of the central tree, which roughly mirror the extension of below ground roots [32], were within the borders of the plot at the onset of the experiment, and none had grown to the outside by the end of the experiment. We therefore assume that the central tree's root system was exclusively under influence of the sediment conditions induced by the experimental treatments of the respective plots (see below).

Plots were randomly assigned to three treatments (crab removal, disturbance control and control; with four replicates each). Possible side effects of the crab removal procedure (see details below) were assessed by the disturbance control plots, where crab removal was only simulated, following the approach of Smith et al. [5]. We added an additional replication per treatment (n = 4), compared to the experimental design of Smith et al. [5]. In each plot, sediment samples were taken to measure a number of abiotic sediment parameters (sediment salinity, sediment organic matter content, reduction potential, CO_2_ efflux rates from the sediment) and one biotic parameter (leaf production estimated by the stipule fall rate); a detailed description of the respective procedures is given below. Three replicate sampling points were randomly chosen inside each plot during each sampling campaign, but in a distance of at least 15 cm away from *U*. *cordatus* burrow entrances and from spots where superficial *R*. *mangle* roots touched the sediment surface. This allowed the investigation of potential larger scale ecosystemic changes beyond the immediate neighbourhood of burrows or roots. The selected parameters were measured at all three points (reduction potential, CO_2_ efflux rate) or at a subset of two (salinity, organic matter).

The experiment ran from 19/11/2011 until 04/11/2012. Sediment salinity, organic matter, CO_2_ efflux rate and reduction potential were measured during eight sampling campaigns. From November 2011 until January 2012 the sampling of the above parameters was conducted monthly, thereafter in intervals of six weeks until April 2012 and after that every two months until November 2012. Stipule fall was assessed by biweekly collection of material in litter traps (24 samplings in total).

### Tidal inundation

Water pressure data loggers (HOBO U20, onset) were employed from 05/03/2012 to 15/03/2012 to determine water levels and calculate inundation frequencies. To extrapolate the data to the entire study period, the pressure data obtained for the 10 days were matched with the tide table for the nearest site from the Brazilian National Oceanographic Database (Banco Nacional de Dados Oceanográficos, BNDO, Fundeadouro de Salinópolis, http://www.mar.mil.br, 2012, 80 km northwest from the study site). Salinity and temperature readings of tidal surface water were taken in the middle of the tidal channel Furo Grande, approximately 300 m away from the experimental plots. Readings were taken in the morning and late afternoon of each sampling day.

### Crab removal

The term “crab removal” rather than of “crab exclusion” is used since no fences or other artificial borders were applied around the experimental plots. This way likely side effects of fencing in this dynamic macrotidal environment, such as changes in sediment deposition and leaf export, were avoided [12,13,19]. *U*. *cordatus* specimens were caught from removal plots by deploying approximately 400 nylon nets (20 cm × 30 cm) per sampling day. The capture technique was modified after a technique called “redinha” (tangle-netting), used illegally by crab fishermen in many other parts of Brazil [34,35]. Each net was fixed to the ground with one cutting of *R*. *mangle* aerial roots (25 cm, long; obtained outside the experimental plots) inserted into the sediment in front a crab burrow. Cuttings were rinsed and dried before applying them to minimize leaching into the sediment. The nets were then slightly pushed into the burrow entrances. The following day, crabs entangled in the nets were counted and the carapace width (cm) of all or every second individual (if there were more than 10) recorded. Due to the activity of crab eating raccoons (*Procyon cancrivorus*) and other predators, captured crabs had frequently been consumed before the tangle-nets could be controlled, indicated by crab remains. These remains were also counted and carapace widths measured, if possible. All survivors were released sufficiently far away from the experimental plots to prevent re-immigration. During the application of nets, care was taken to minimize sediment disturbance, e.g. by using firm stilt roots as walkways. Crab removal started with the first sampling campaign in November 2011 and was conducted biweekly for 3–6 days (3 days sampling during crab catching campaigns and 6 days during campaigns for sampling environmental parameters only) for each plot during neap tides over one year. Crab removal was conducted during neap tides, because crabs are more active then, and close their burrows less frequently than during spring tides [36]. Capture success was calculated for each removal plot by dividing the number of captured living crabs and carapace remains by the number of installed nets and the number of capture days (crabs d^-1^ net^-1^). Additional manual removal of crabs moving around freely inside the plots was necessary during mass mate searching events during spring tides [37]. These events occurred after new moon in January 2012 and after full moon in February and March 2012. Crab burrow density (burrows m^-2^) was monitored every 4–5 weeks over the entire study period by counting closed and open burrows in always the same two 1 m × 13 m subplots per removal plot. For the disturbance control plots the applied capture technique was simulated by pushing the mangrove cuttings without nets into the ground, followed by removal of the cutting, and stressing the tree roots by walking over them to a similar extent as in the removal plots.

### Organic matter content and salinity of the sediment

Two sediment cores were taken per plot and sampling campaign with a peat sampler (Eijkelkamp) of 50 cm length and 6 cm diameter. Subsamples of the extracted sediment were taken at core depths of 1, 5, 10, 20, 30, 40, and 50 cm, filled into plastic vials and stored at temperatures *≤* 0°C until further processing. Samples were homogenized and divided into two portions. One portion was used for the gravimetrical determination of the water content of the sediment through weight loss by drying at 104°C. The organic matter content of the dry sample was obtained subsequently through weight loss by combustion at 450°C. The second portion was used to analyse sediment salinity. Two grams of the sediment were mixed with 10 ml of distilled water and shaken for 24 h on a mechanical shaker (MA136, Marconi). Afterwards, the salinity of the sediment extract was measured with a WTW TetraCon 325 conductivity meter connected to a WTW multi-parameter instrument (340i). Sediment salinity was calculated based on the previously measured original water content of the respective subsample [38].

### CO_2_ efflux rate of the surface sediment

Six CO_2_ efflux rate measurements of the surface sediment were performed in each of the twelve plots at each sampling date. At each of the three sampling points (see above), two PVC collars of 20 cm diameter were inserted several centimetres into the sediment void of visible roots, *U*. *cordatus* burrows and mostly also void of burrows of other crab species. These two collars were handled as replicates for one sampling point. A distance of 40–50 cm was maintained between the two collars at each sampling point, large enough to ensure that the disturbance created by the insertion of one collar into the sediment would not affect the sediment of the other collar and small enough to represent the sampling point. To avoid any influence of CO_2_ release due to the insertion of the collars, measurements were started 1 h after the installation. An opaque respiration chamber was connected to a CO_2_/H_2_O infrared gas analyser (LI-8100A, LI-COR, Biosciences) and fitted on top of the PVC collar. The CO_2_ concentration inside the chamber was recorded for 2 min. The measurement was repeated four times per collar. Between replicates, the chamber was opened for 25 s to release the accumulated CO_2_. Sediment temperature was measured outside the collar at 2 cm sediment depth by thermocouple (OMEGA Engineering). The CO_2_ efflux rates were calculated [38], assuming a linear increase in CO_2_ concentration over time. A correction for changing sediment temperature was applied.

### Reduction potential

Three sediment cores were taken in each plot per sampling date at the three sampling points. Redox potential (± 1.0 mV), pH (± 0.1) and temperature (± 0.1°C) were measured within the sediment cores immediately after their extraction at 1, 5, 10, 20, 30, 40, and 50 cm depth with a Sartorius ORP (redox) combination electrode and a WTW Sentix 41 pH-electrode connected to a WTW portable meter (Multi 340i), respectively.

As indicator of the reduction force of a reduction system, the rH value was calculated including the redox potential, temperature and pH value of each measurement [39]. rH values range between 0 (strongly reducing conditions) and 42 (strongly oxidizing conditions).

### Stipule production

Stipule fall of *R*. *mangle* trees is related to the unfolding of new pairs of leaves. It can therefore be used as an indicator for the leaf production of these trees [40–42]. Stipule fall in the genus *Rhizophora* is known to be influenced by sediment characteristics [40–42] and was shown to respond to changing sediment conditions within one year [5].

Litter of the central tree in each plot was sampled with two litter traps (0.25 m^2^ each) fixed to the stem at 50 cm horizontal distance in 5–7 m height to ensure autochthonous litter. Traps were emptied biweekly. Stipules were separated from other litter components and dried at 104°C to constant weight (g). Stipule dry matter from both collectors was pooled and stipule fall rates (g m^-2^ d^-1^) calculated.

### Statistical analyses

The statistical analyses were carried out following the protocols for data exploration and analysis of Zuur et al. [43,44] using the statistical programming environment R [45] with the packages “nlme” [46], “mgcv” [47], “lattice” [48] and “ggplot2” [49]. Presented values are shown as mean ± standard error (se). Before further analyses, data were checked for outliers (Cook's distance), which were removed, if necessary. All data for the analysis are available in the supporting information (S1 File).

Carapace widths of the captured crabs at each plot were analysed for differences over time with a one-way ANOVA. Linear mixed-effects models (LME) and generalized additive mixed-effects models (GAMM) [47,50,44,51] were used to model individual response variables (sediment salinity, organic matter content, CO_2_ efflux rate and reduction potential/rH) in relation to different treatments, time and their interaction effect. In some models (including sediment salinity, organic matter content and rH), sediment depth was considered as an additional covariate. Plot and sampling points within plots (when appropriate) were used as random terms to account for the nested structure of the experimental design. When trends over sediment depth or time were not linear, these covariates were set as categorical covariates. To find the optimal fixed terms, stepwise backward model selection was used based on the maximum likelihood ratio test (ML) and/or the Akaike Information Criterion (AIC). When an interaction was part of the final model, all participating main factors were automatically retained. The validity of the models was checked by examining diagnostic plots of residual versus fitted values and residuals versus covariates. Independence was examined by plotting residuals versus time. Final models were presented with the restricted maximum likelihood estimation method (REML).

Stipule data were analysed with a GAMM model for differences among treatments over time. GAMM's are non-parametric regression models and allow, in the case of the stipule data, for nonlinear trends over time with a smoothing function for the predictor variable time.

## Results

### Inundation levels

**Fig 1:**
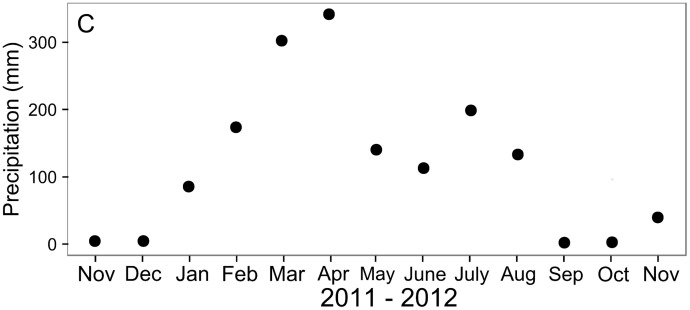
Precipitation data of the study area. Total monthly precipitation (mm) recorded at the weather station in Tracuateua, 50 km southwest from the study site (INMET, 2013). Data sets are from November 2011 until November 2012.

All twelve experimental plots had similar inundation levels and were flooded during high tide on 131 days out of the 355 days of the study period. This corresponded to a flooding frequency of 14–19 days per month. Surface water salinity at the Furo Grande varied from 22.8 to 36.9 during the twelve months. Lowest salinities were recorded during periods of high rainfall (Fig 1). Surface water temperatures ranged between 27.1°C and 30.5°C.

### Crab removal

It proved logistically feasible to set up to approximately 400 nylon nets per sampling day and per removal plot, which initially covered around 50% of all *U*. *cordatus* burrows and, at six months of the experiment, around 90–100% of all *U*. *cordatus* burrows. In total, 4866 crabs were caught during the one year study, including live crabs (2872), and remains of dead crabs (1994). Additional crab parts scattered by predators within plots of further 1563 captured crabs were counted, but not included in the capture success calculation for a more conservative estimate. During mass mate searching events 844 crabs were additionally caught by hand inside the removal plots. Capture success varied over the year from 0 to 0.2 crabs d^-1^ net^-1^ with a higher success during the mass mate searching events between January and March 2012 (S1 Fig). Crab burrow density in the removal plots slowly decreased until stabilizing more or less towards the end of the experiment. Burrow density inside the removal plots decreased on average to 52% of the initial number (Table 1, data from other plots and samplings are listed in in the supporting material in S1 Table). The carapace width of the captured *U*. *cordatus* specimens decreased over time in removal plot 1 (F-value = 50.4, df = 1, *p*-value < 0.001) and 3 (F-value = 38.9, df = 1, *p*-value < 0.001), showing that the larger animals (the more efficient bioturbators) were constantly removed. However, it did not differ over the year in removal plot 2 (F-value = 2.6, df = 1, *p*-value = 0.1) and 4 (F-value = 0.3, df = 1, *p*-value = 0.6) (Table 2).

**Table 1:** Crab burrow density. *U*. *cordatus* burrow density (burrows m^-2^) inside the four removal plots for the first and last sampling. Decrease in burrow density from the first until the last sampling is given in %. Data from the other plots and sampling occasions are listed in the supporting material (S1 Table).

Removal plot	1. sampling	8. sampling	Decrease in %
1	4.7	2.1	55.3
2	6.7	3.9	41.8
3	3.1	0.5	83.9
4	4.4^a^	3.2	27.3

^a^ Crab burrow density of plot 4 was estimated one month after the first sampling; decrease in % may thus be underestimated.

**Table 2:** Mean carapace width of *U*. *cordatus*. Mean carapace width ± standard deviation of captured crabs and carapace remains of the first and last sampling. Sample size is given in the brackets. The change of the carapace width from the first to the last sampling is given in % and its statistical significance is given in the brackets (*p*).

Removal plot	1. sampling	8. sampling	Change in % (*p*-value)
1	6.5 ± 0.8 (35)	5.2 ± 1.0 (25)	- 20 (*p* < 0.001)
2	5.1 ± 0.7 (35)	5.4 ± 0.9 (30)	+ 6 (*p* = 0.1)
3	6.1 ± 0.9 (18)	5.7 ± 1.0 (25)	- 7 (*p* < 0.001)
4	5.0 ± 0.7 (50)	5.1 ± 1.0 (43)	+ 2 (*p* = 0.6)

### Sediment parameters

**Fig 2:**
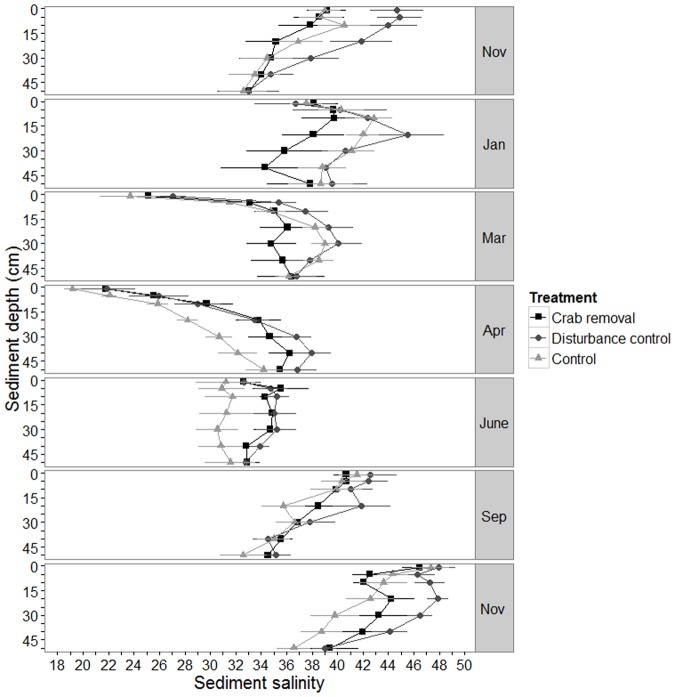
Measured sediment salinity. Mean sediment salinity ± standard error (se) over sediment depth (cm) in crab removal plots, disturbance control plots and control plots (see legend for symbols). The data of seven sampling campaigns between November 2011 and November 2012 are plotted. Values for the second sampling in December are missing because of technical problems.

No consistent difference between treatments in respect to sediment salinity across depth and time were detected. In months with significant rainfall, salinity was lowest near the sediment surface and increased gradually with depth (Fig 2, March and April). In the dryer months salinities tended to be high over the whole sediment depth range. This relationship was reflected by the final LME model including a three way interaction (L. Ratio = 26.2, df = 12, *p*-value = 0.01, S2 File).

**Fig 3:**
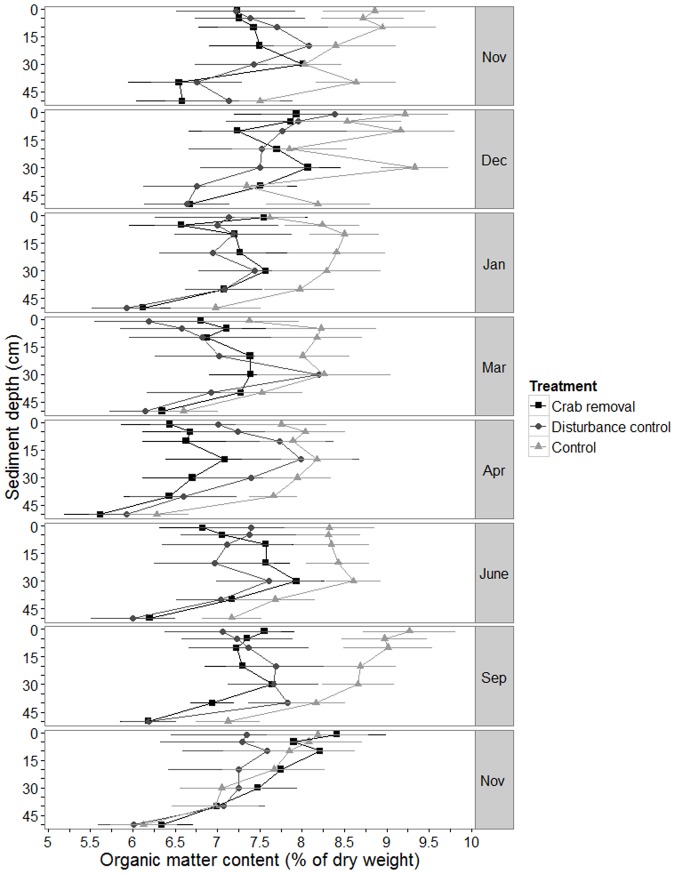
Measured organic matter content. Mean organic matter content ± standard error (se) (% of dry mass) over sediment depth (cm) in crab removal plots, disturbance control plots and control plots (see legend for symbols). The data of eight sampling campaigns between November 2011 and November 2012 are plotted.

Treatments for the organic matter content did not differ among each other over time (Fig 3) (interaction term treatment × time was not significant, L. Ratio = 2.0, df = 2, *p*-value = 0.4, S3 File). However, organic matter content was generally lowest at the greatest depth. The form of the organic matter-depth curves differed slightly between treatments at each sampling campaign (interaction term sediment depth × treatment: L. Ratio = 8.7, df = 2, *p*-value = 0.01, S2 Table); control values were higher than those of the other two treatments for most sampling date/depth combinations (Fig 3). Changes also occurred among sampling campaigns as reflected by the significant interaction term sediment depth × time included in the final model (L. Ratio = 6.2, df = 1, *p*-value = 0.01, S3 File).

**Fig 4:**
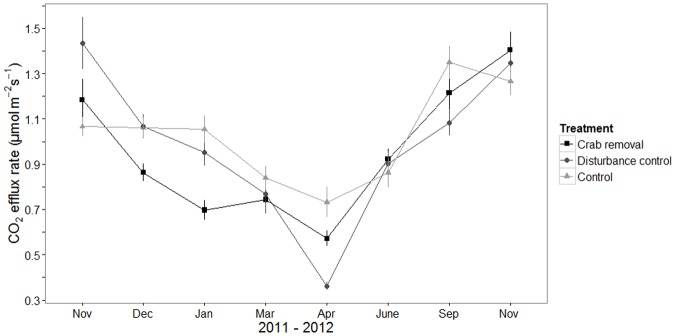
Measured CO_2_ efflux rate. Mean CO_2_ efflux rate (μmol m^-2^ s^-1^) ± standard error (se) in crab removal plots, disturbance control plots and control plots (see legend for symbols). The data of eight sampling campaigns between November 2011 and November 2012 are plotted.

The CO_2_ efflux rate of the surface sediment showed the same seasonal trend in all treatments, with lowest CO_2_ efflux rates in the peak wet season (Fig 4). Only the variable time was significant (L. Ratio = 100.2, df = 7, *p*-value < 0.001, S4 File).

**Fig 5:**
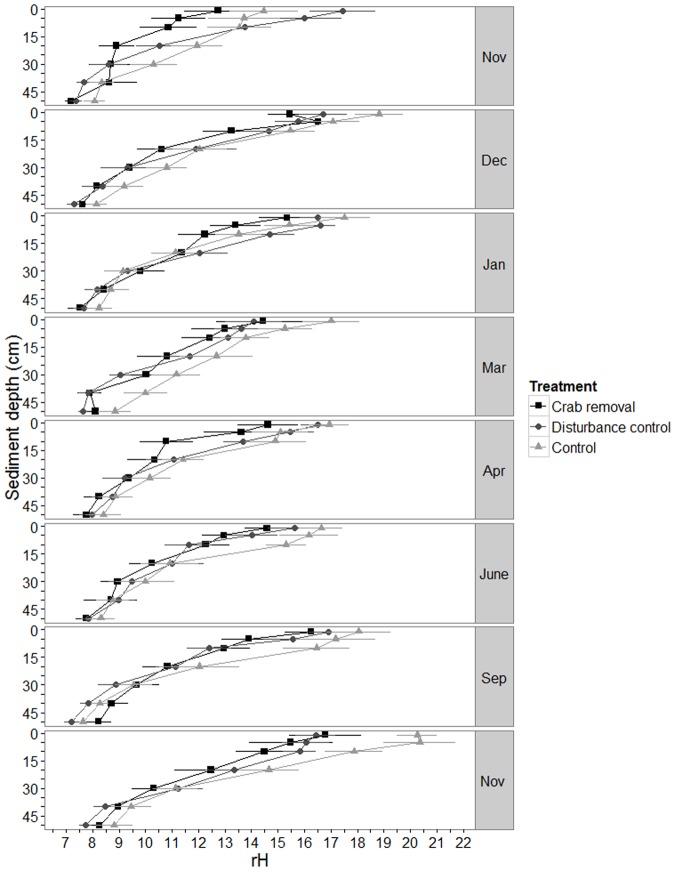
Measured rH values. Mean rH ± standard error (se) over sediment depth (cm) in crab removal plots, disturbance control plots and control plots (see legend for symbols). The data of eight sampling campaigns between November 2011 and November 2012 are plotted.

rH values in all treatments decreased with depth during all sampling campaigns. However, no distinct difference among treatments evolved over time (Fig 5). The final model retained a three-way interaction (L. Ratio = 29.04, df = 12, *p*-value = 0.004, S5 File), indicating that the specific form of the rH-depth curves was not consistent over all treatment and sampling dates.

### Stipule production

**Fig 6:**
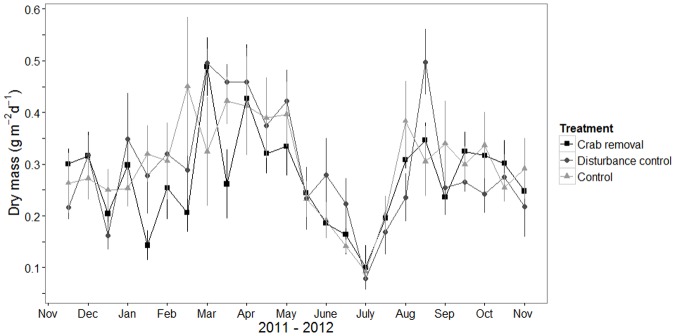
Measured stipule fall rate. Mean stipule fall rate in dry mass ± standard error (se) (g m^-2^ d^-1^) for *R*. *mangle* trees in crab removal plots, disturbance control plots and control plots (see legend for symbols). The data of 24 biweekly sampling campaigns between November 2011 and November 2012 are plotted.

Stipule fall rate did not differ significantly between treatments (F-value = 0.3, df = 2, *p*-value = 0.7, S6 File). However, it showed a distinct bimodal temporal pattern, therefore the inclusion of a smoothing function for the variable time improved the model (F-value = 16.9, df = 7.9, *p*-value < 0.001, S6 File). Peaks appeared in March-April and in August 2012 (Fig 6).

## Discussion

None of the studied response parameters was affected by the decrease in *U*. *cordatus* burrow numbers in the one year removal experiment, hence all initially stated hypotheses have to be rejected. As in the crab removal study of Smith et al. [5] in Australia, crabs were not completely removed from the exclusion plots in our experiment (we simulated a distinctly decreased fishing pressure rather than catastrophic mass mortality), but their numbers significantly decreased by 52% of the initial overall burrow density of 3.7–6.7 burrows^-1^ m^-2^. We increased the number of replication by a factor one compared to the Australian study (a further increase of plot numbers was logistically unachievable) and due to the inclusion of a disturbance control treatment in our experimental design, we can exclude that the findings are procedural artefacts. In fact, here we show for the first time that the mangrove ecosystem of the North Brazilian Amazon may be resilient to reduced *U*. *cordatus* numbers within a time span of one year, at least regarding the measured parameters.

### Salinity

The rational of our initial hypothesis that large *U*. *cordatus* burrows would significantly desalinise the sediment was based on findings of studies of other species [12,52,53]. For example, Smith et al. [12] found an increase in sediment salinity after experimental reduction of the number of *Uca* spp. burrows. However, in contrast to their study which was performed in relatively open plots with small mangrove seedlings, our study was performed in a mature, closed-canopy *R*. *mangle* forest (compare Table 3). The lack of salinity reduction through *U*. *cordatus* burrows suggests that the amounts of salt removed by flushing of *U*. *cordatus* burrows are insignificant compared to the amount of salt accumulated by the extensive root systems of the central mangrove trees during water uptake [53–55]. Furthermore, burrows with one opening, typical for *U*. *cordatus* [56] as well as for *Uca* spp., may not be as efficient as desalinators as the burrows with multiple openings of many sesarmid crabs in the IWP which allow a flow through of tidal water between openings [57–60].

**Table 3:** Comparison of exclusion/removal experiments with burrowing crabs from the literature.

Study	This study	Smith et al. 1991 [5]	Smith et al. 2009 [12]	Dye & Lasiak 1986 [13]	Thomas & Blum 2010 [19]
**Habitat**	Mangroves	Mangroves	Restored coastal marsh	Salt marsh	Salt marsh
**Tide**	semidiurnal	na	semidiurnal	semidiurnal	na
**Tidal amplitude**	3–5 m	na	1 m	1.5 m	0.25 m
**Daily flooding**	Only during spring tide	na	Not daily	na	29 times in one year
**Study site**	High intertidal	Low intertidal	na	Mid tide level	na
**Tree density in plots**	6–18	52–81	1	0	na
**Tree species in plots**	*Rhizophora mangle*	*Rhizophora apiculata*, *R*. *stylosa*, *R*. *lamarckii*	*Languncularia racemosa*	-	*Spartina alterniflora*
**Number of plots**	12	9	15	5	12
**Size of plots**	13 m × 13 m	15 m × 15 m	1 m × 1 m	Exclosure: 10–20 cm diameter PVC pipes, control: 0.25 m^2^	Exclosure 1.5 m^2^, others 1 m^2^
**Tree height**	10–14 m	na	ca. 34–65 cm	-	-
**Crab species**	*Ucides cordatus*	*Sesarma* spp.	*Uca* spp.	*Uca vocans*, *U*. *polita*	*Uca pugnax*
**Crab catching**	Nylon nets	Pitfall traps	By hand, enclosure	Exclosure	Exclosure
**Removed crabs**	4866	over 1500	na	na	na
**Time of catching**	Biweekly for 3–6 days	constant	Before experiment started	na	Before experiment started
**Removal efficiency**	In average 52%	70–80%	na	na	na
**Sampling time**	1 year	1 year	11 months	14 days	18 months
**Treatments**	Removal, disturbance control, control	Removal, disturbance control, control	Exclusion, control	Exclusion, control	Exclusion, adding artificial burrows, crabs naturally (not) present
**Effects due to crab removal or exclusion**	none	Soil sulphide and ammonium concentration increased	Height, trunk diameter and leaf production decreased	Abundance of meiobenthos increased 2 to 5-fold	Decrease in soil redox potential
		Decrease of forest growth (by stipule fall)	Increase of interstitial water salinity		Decrease in sediment decomposition
		Less reproductive output (by mature propagule fall)	Decreased the oxidation-reduction potential of the lower organic sediments		Accumulation of carbon in the sediment

### Organic matter content and CO_2_ efflux rate

Our hypothesis of decreased sediment organic matter storage due to removal of *U*. *cordatus* was based on the fact that these crabs are the dominant litter feeder in Brazilian mangrove forests; as such, the animals retain litter material in the mangrove forest that would otherwise be flushed away by the tides [8,9]. In addition to their (sloppy) feeding at the sediment surface, the crabs carry litter into their burrows where it is often only partially consumed [36,61]. A removal of crabs should therefore lead to an increase in sediment organic matter content. However, *U*. *cordatus* does not only accumulate organic matter, but at the same time also facilitates organic matter processing by other organisms, leading to a decrease in organic matter stock. Enhanced organic matter decomposition can be increased under drier conditions (e.g. low tide, dry season), when burrow walls are more oxidized due to contact with atmospheric oxygen. Consequently, carbon oxidation is facilitated by the presence of burrows, resulting in diminishing sediment organic matter [1,62]. A decreasing number of crabs would therefore affect both (antagonistic) processes and could potentially result in a zero net change. In addition, competition for leaf litter among crabs is strong [8]. Lower crab densities (i.e. inside the removal plots) may therefore allow the remaining crabs to increase their per capita food uptake, allowing them to process the same amount of leaf litter as in a situation with higher crab numbers.

Regarding sediment CO_2_ efflux rates we assumed that crab removal would lead to a decrease in this parameter. Sediment CO_2_ efflux rates reflect the activity of microbes, and a reduced crab feeding activity would lead to a decrease in substrate availability for these organisms. However, since no changes in the organic matter content of the sediment occurred, it is not surprising that sediment CO_2_ efflux rates did also not change.

### rH

Crab burrows may influence the reduction state of the sediment. Pülmanns et al. [63] showed such an effect at least for the immediate neighborhood of *U*. *cordatus* burrow walls. In a North-Eastern Brazilian area with burrow densities much higher than at our study site (12 ± 3 burrows m^−2^ versus 6.7 burrows m^−2^ [64]), *U*. *cordatus* bioturbation led to more oxidizing conditions. However, our rH results do not support a general, i.e. far-reaching effect of the burrows on sediment reduction state, probably due to the limited reach of aeration effects at individual burrows [63] in combination with the relatively low burrow densities (also in our control plots). Under these conditions, overlap between oxidized zones around burrows is minimal.

Several other studies focusing on fiddler crabs, with manifold higher burrow densities (60 to more than 200 burrows m^-2^), also recorded substantial changes in the reduction potential for the upper sediment layer in bioturbated areas [15,65–69]. However, most authors do not report the distance between sampling points and the nearest burrows, making it difficult to compare their data with ours.

### Stipule production

Since none of the measured sediment parameters changed with crab removal, it is not surprising that stipule fall rate did not change in the removal plots.

Experimental removal of mostly sesarmid mangrove crabs (which are generally much smaller than *U*. *cordatus*) resulted in a distinct decrease in stipule fall rate during a one year study period in Australia [5]. In contrast to our one year experiment, sediment conditions in the Australian crab removal study changed inside the exclusion plots (increased concentration of sulfide and ammonium) as well as stipule fall [5]. It remains unclear why stipule fall in the Australian mangrove ecosystem was affected by the reduction of burrow density, while in North Brazilian it was not. This is even more intriguing since the total number of caught crabs in our study was more than threefold higher than that of the Australian study (Brazil: in total 4866 *U*. *cordatus* caught with nets, Australia: approximately 1500 crabs—mostly *Sesarma messa* and *Sesarma semperi longicristatum*—caught with pitfall traps), and our design included four replicate plots, compared to only three in the Australian study. One reason for this outcome could be that our plots contained 6–18 relatively large trees each, whereas the Australian plots contained 52–81 smaller trees. Younger trees with smaller root system extension may react faster to changes in sediment characteristics than more mature trees with high root biomass. Furthermore, the location of the study site along the tidal gradient differed between the two sites. Our study was conducted in the high intertidal, whereas Smith III et al. [5] worked in the lower intertidal which was probably more frequently inundated. Thus, in the Australian system, regular tidal flushing of crab burrows is an important factor amplifying the role of burrows in contrast to the situation in our study, where the effects of (rare) flushing of the burrows may be too insignificant to influence sediment characteristics within one year.

### Seasonal effects

In contrast to the lack of consistent differences in any of the measured parameters between crab removal and both control treatments, most parameters exhibited distinct seasonal changes. Precipitation is an important abiotic factor influencing sediment salinity conditions in mangrove forests [70,71]. Changes in sediment salinity can influence growth and phenology of *R*. *mangle* trees, and tree growth and flower bud production is enhanced during the wet season [72–74]. This agrees with the observed highest stipule fall rates in our plots from March to April when sediment salinities were lowest. Precipitation does not only affect salinity, but can, independently from the tidal cycle, saturate the sediment with water, creating less oxidized conditions over extended time intervals. This may lead to sulfate reduction in the upper sediment layer [70]. In our study, slightly lower rH values were recorded at the sediment surface during the wet season (Fig 2). Consequently, waterlogged and more anoxic sediment conditions in the upper sediment layers may have led to reduced carbon oxidation rates, resulting in lower CO_2_ release (Fig 1) as observed elsewhere [1,70,75]. Overall, our results suggest that seasonal changes in precipitation are more important drivers for the measured parameters than the *U*. *cordatus* burrows at the given low natural crab density at our macrotidal study site.

## Conclusion

At our Amazonian mangrove study site, all measured parameters remained unaffected by the artificial removal of more than 4866 *U*. *cordatus* over one year from four 13 m × 13 m plots. We substantially reduced the initial burrow density by more than 50% and thus simulated a clear substantial increase in fishery or pathogen pressure. However, during our one year study the pronounced seasonal changes in precipitation had a much stronger influence on the measured parameters than the crabs’ bioturbation and leaf litter feeding. An experimental duration of several years could yield different results, due to potential accumulation of (subtle) effects of reduced crab numbers. A different experimental outcome than ours could also be thinkable for areas with higher initial crab densities and/or less pronounced rainfall during the rainy season than in Amazonian. Comparative removal studies involving the same crab species in different environmental contexts would further improve our understanding of the relative importance (and plasticity) of abiotic versus biotic factors as drivers of mangrove ecosystem functioning.

## Supporting Information

S1 Fig *Ucides cordatus* capture success. (PDF)

S1 File Data set for statistical analyses. (XLSX)

S2 File Final linear mixed-effects model of the sediment salinity. (PDF)

S3 File Final linear mixed-effects model of the organic matter content. (PDF)

S4 File Final linear mixed-effects model of the CO_2_ efflux rate. (PDF)

S5 File Final linear mixed-effects model of the rH value. (PDF)

S6 File Final linear mixed-effects model of the stipule fall rate. (PDF)

S1 Table List of *Ucides cordatus* burrow densities in plots. (PDF)
