# Supplementary material for: Mangrove Crab Ucides cordatus Removal Does Not Affect Sediment Parameters and Stipule Production in a One Year Experiment in Northern Brazil
Source: PLoS One. 2016 Dec 1;11(12):e0167375. doi: 10.1371/journal.pone.0167375 (PMC5131973; doi:10.1371/journal.pone.0167375)

S1 Figure. ***Ucides cordatus*** **capture success.** Mean crab capture success ± standard error (se) (crabs d^{-1} net^{-1}) for the removal plots from November 2011 until November 2012. Crab capture was conducted biweekly during neap tides for 3-6 days. Crab capture success was calculated considering both living animals and carapace remains.

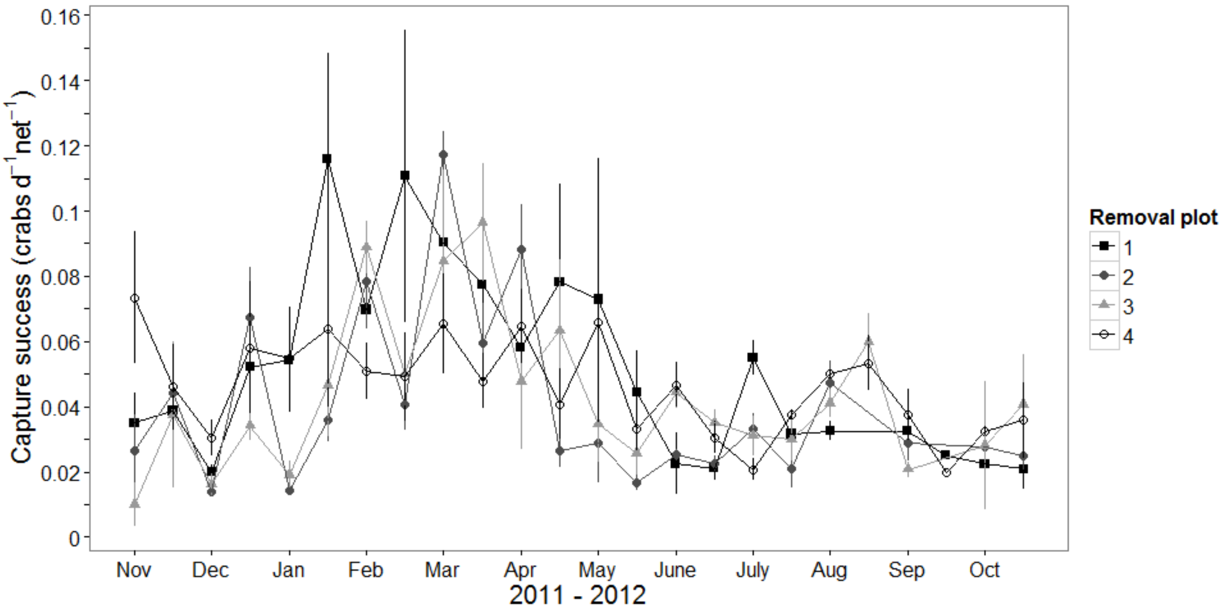

Supplement: S1 Fig — (PDF) [file pone.0167375.s001.pdf]
