# Supplementary material for: Mangrove Crab Ucides cordatus Removal Does Not Affect Sediment Parameters and Stipule Production in a One Year Experiment in Northern Brazil
Source: PLoS One. 2016 Dec 1;11(12):e0167375. doi: 10.1371/journal.pone.0167375 (PMC5131973; doi:10.1371/journal.pone.0167375)
Supplement: S2 File — (PDF) [file pone.0167375.s003.pdf]

S2 File. Final linear mixed-effects model of the sediment salinity.

The final optimal model was selected after a stepwise backwards model selection using the likelihood ratio test:

$$Sediment\ salinity_{ip} \sim \alpha + Treatment_{icp} + Sediment\ depth_{icp} + Time_{icp} + Treatment_{icp} \times Sediment\ depth_{icp} + Treatment_{icp} \times Time_{icp} + Sediment\ depth_{icp} \times Time_{icp} + Treatment_{icp} \times Sediment\ depth_{icp} \times Time_{icp} + a_p + a_{c/p} + \varepsilon_{icp}, \quad \varepsilon_{icp} \sim N(0,\sigma^2)$$

Sediment salinity_{icp} is the observation *i* for each sediment core *c* at each plot *p*, where *c* runs from 1 to 3, *p* from 1 to 12 and *i* is the observation for each core at the different sites that goes from 1 to 7 (the number of samplings over time).The final model above means that Sediment salinity is modelled as a function of Treatment, Sediment depth, Time, and all their two and three way interactions. Treatment and Time are a categorical covariate and Sediment depth is continuous. Time was set as categorical covariate, because differences between points of time were not linear. The terms a_p and $a_{c/p}$ are random effects representing the between-plot and between-core variation and are significant (L. Ratio = 71.7, df = 1, *p*-value < 0.001, nested term: L. Ratio = 449.1, df = 1, *p*-value < 0.001). The unexplained variance ε_{ics} is assumed to be normally distributed with mean 0 and variance σ^2 considered for each sediment depth *d* separately. The intercept of the model is represented with α.
