# Supplementary material for: Mangrove Crab Ucides cordatus Removal Does Not Affect Sediment Parameters and Stipule Production in a One Year Experiment in Northern Brazil
Source: PLoS One. 2016 Dec 1;11(12):e0167375. doi: 10.1371/journal.pone.0167375 (PMC5131973; doi:10.1371/journal.pone.0167375)
Supplement: S4 File — (PDF) [file pone.0167375.s005.pdf]

S4 File. Final linear mixed-effects model of the CO_2 efflux rate.

The final optimal model was selected after a stepwise backwards model selection using the likelihood ratio test:

$$CO_2\ efflux\ rate_{up} \sim \alpha + Time_{icp} + a_p + a_{u/p} + \varepsilon_{iup}, \qquad \varepsilon_{iup} \sim N(0,\sigma^2)$$

CO_2 efflux $rate_{iup}$ is the observation *i* for each sediment sampling unit *u* at each plot *p*, where *u* runs from 1 to 4, *p* from 1 to 12 and *i* is the observation for each sampling units at the different plots that goes from 1 to 8 (the number of samplings over time). The final model above means that CO_2 efflux rate is modelled as a function of Time. Time is a categorical covariate. The terms a_p and $a_{u/p}$ are random effects representing the between-plot and between-core variation and are significant (L. Ratio = 26.3, df = 1, *p*-value < 0.001, nested term: L. Ratio = 1968.8, df = 1, *p*-value < 0.001). The unexplained variance ε_{iup} is assumed to be normally distributed with mean 0 and variance σ^2. The intercept of the model is represented with α.
