# Supplementary material for: Mangrove Crab Ucides cordatus Removal Does Not Affect Sediment Parameters and Stipule Production in a One Year Experiment in Northern Brazil
Source: PLoS One. 2016 Dec 1;11(12):e0167375. doi: 10.1371/journal.pone.0167375 (PMC5131973; doi:10.1371/journal.pone.0167375)
Supplement: S6 File — (PDF) [file pone.0167375.s007.pdf]

S6 File. Final linear mixed-effects model of the stipule fall rate.

The final optimal model was selected after a stepwise backwards model selection using the likelihood ratio test:

$$Stipule\ fall\ rate_{ip} \sim \alpha + s(Time)_{ip} + a_p + \varepsilon_{ip}, \quad \varepsilon_{ip} \sim N(0,\sigma^2)$$

Stipule fall rate$_{ip}$ is the observation *i* for each plot *p*, where *p* runs from 1 to 12 and *i* is the observation within a plot which goes from 1 to 24 (the number of samplings over time).The final model above means that Stipule fall rate is modelled as a function of Time. Time is a continuous variable. The notation *s* stands for smoothing function of time. The terms a_p and $a_{u/p}$ are random effects representing the between-plot and between-core variation and are significant (L. Ratio = 15.0, df = 1, *p*-value < 0.001). The unexplained variance ε_{ip} is assumed to be normally distributed with mean 0 and variance σ^2. The intercept of the model is represented with α.
