# Supplementary material for: Mangrove Crab Ucides cordatus Removal Does Not Affect Sediment Parameters and Stipule Production in a One Year Experiment in Northern Brazil
Source: PLoS One. 2016 Dec 1;11(12):e0167375. doi: 10.1371/journal.pone.0167375 (PMC5131973; doi:10.1371/journal.pone.0167375)
Supplement: S1 Table — (PDF) [file pone.0167375.s008.pdf]

S1 Table. List of *Ucides cordatus* burrow densities in plots. *U. cordatus* burrow density (burrows m^{-2}) samples were taken in the high intertidal zone during neap tides at the Furo Grande, Caeté estuary, North Brazil. Data complete for removal plots, but selectively measured for the remaining treatments. Data in grey shaded columns correspond to the first and last parameter samplings. Other burrow counts were taken during the crab catching campaigns between parameter samplings.

Removal plots	Nov 2011	Dec 2012	Jan 2012	Feb 2012	Apr 2012	May 2012	June 2012	July 2012	Aug 2012	Sept 2012	Oct 2012	Nov 2012
1	4.7	2.2	2.5	2.3	2.8	2.0	2.2	2.5	3.5	3.2	2.9	2.1
2	6.7	5.3	3.1	2.5	3.7	2.6	2.2	4.3	4.0	2.8	3.5	3.9
3	3.1	1.8	1.9	1.2	2.7	1.5	1.3	1.2	1.3	1.3	1.4	0.5
4	na	4.4	4.6	3.0	2.0	2.4	2.7	3.1	3.7	3.4	3.2	3.2
**Control plots**												
1	na	na	na	na	na	na	na	na	na	3.3	3.6	3.3
2	na	na	na	na	na	na	na	na	na	3.8	4.8	3.7
3	na	na	na	na	na	na	na	na	na	2.4	5.6	3.5
4	na	na	na	na	na	na	na	na	na	2.6	4.2	3.5
**Disturbance control plots**												
1	na	na	na	na	na	na	na	na	na	2.6	4.5	3.8
2	na	na	na	na	na	na	na	na	na	3.1	4.5	3.3
3	na	na	na	na	na	na	na	na	na	3.5	4.4	4.4
4	na	na	na	na	na	na	na	na	na	2.8	2.3	2.0
